# Copper boosts the biostimulant activity of a vegetal-derived protein hydrolysate in basil: morpho-physiological and metabolomics insights

**DOI:** 10.3389/fpls.2023.1235686

**Published:** 2023-08-24

**Authors:** Youssef Rouphael, Petronia Carillo, Michele Ciriello, Luigi Formisano, Christophe El-Nakhel, Paola Ganugi, Andrea Fiorini, Begoña Miras Moreno, Leilei Zhang, Mariateresa Cardarelli, Luigi Lucini, Giuseppe Colla

**Affiliations:** ^1^ Department of Agricultural Sciences, University of Naples Federico II, Portici, Italy; ^2^ Department of Environmental, Biological and Pharmaceutical Sciences and Technologies, University of Campania “Luigi Vanvitelli”, Caserta, Italy; ^3^ Department for Sustainable Food Process, Università Cattolica del Sacro Cuore, Piacenza, Italy; ^4^ Department of Sustainable Crop Production, Università Cattolica del Sacro Cuore, Piacenza, Italy; ^5^ Department of Agriculture and Forest Sciences, University of Tuscia, Viterbo, Italy; ^6^ CRAST Research Centre, Università Cattolica del Sacro Cuore, Piacenza, Italy

**Keywords:** vegetal protein hydrolysates, sustainable agriculture, primary metabolism, oxidative stress, metabolomics, photosynthetic performance, ionomics

## Abstract

In addition to be used as a plant protection agent, copper (Cu) is also an essential micronutrient for plant growth and development. The bioavailability of Cu in agricultural systems can be limited due to its specific physical–chemical characteristics, leading to imbalances in plant production. To address this issue, an experimental trial was conducted on Genovese basil (*Ocimum basilicum* L.) in protected conditions to comparatively evaluate the effects of a vegetable protein hydrolysate (VPH), free Cu and Cu complexed with peptides and amino acids of vegetal origin (Cu and Cu-VPH, respectively), and a combination of VPH and Cu-VPH (VPH+Cu-VPH). The study showed that the combined application of VPH+Cu-VPH led to a significant average increase of 16.3% in fresh yield compared to the untreated Control and Cu treatment. This finding was supported by an improved photosynthetic performance in A_CO2_ (+29%) and Fv/Fm (+7%). Furthermore, mineral analysis using ICP OES demonstrated that Cu and Cu-VPH treatments determined, on average, a 15.1-, 16.9-, and 1.9-fold increase in Cu in plant tissues compared to control, VPH, and VPH+Cu-VPH treatments, respectively. However, the VPH+Cu-VPH treatment induced the highest contents of the other analyzed ions, except for P. In particular, Mg, Mn, Ca, and Fe, which take part in the constitution of chlorophylls, water splitting system, and photosynthetic electron transport chain, increased by 23%, 21%, 25%, and 32% compared to respective controls. Indeed, this improved the photosynthetic efficiency and the carboxylation capacity of the plants, and consequently, the physiological and productive performance of Genovese basil, compared to all other treatments and control. Consistently, the untargeted metabolomics also pointed out a distinctive modulation of phytochemical signatures as a function of the treatment. An accumulation of alkaloids, terpenoids, and phenylpropanoids was observed following Cu treatment, suggesting an oxidative imbalance upon metal exposure. In contrast, a mitigation of oxidative stress was highlighted in Cu-VPH and VPH+Cu-VPH, where the treatments reduced stress-related metabolites. Overall, these results highlight an interaction between Cu and VPH, hence paving the way towards the combined use of Cu and biostimulants to optimize agronomic interventions.

## 1 Introduction

A balance of nutrients, especially between macro- and micronutrients, is essential to ensure plant health and the sustainability of agricultural productions (Souri et al., 2017). Despite being required in smaller amounts than primary nutrients, micronutrients play a crucial role in plant metabolism and physiology. Accordingly, plants must efficiently control their mobilization, absorption, distribution, and storage (Hänsch and Mendel, 2009). Among micronutrients, Cu, a redox-active transition metal, plays a fundamental role in various physiological and biochemical processes (Kaewchangwat et al., 2017). At optimal levels (between 5 and 30 mg kg^–1^), this microelement participates in primary metabolic processes (mitochondrial respiration, electron transport chain, and photosynthesis), lignin biosynthesis, hormone signaling, and response to oxidative stress (Mir et al., 2021). However, since the 1880s, the role of Cu has been relegated to an essential active ingredient in fungicidal and bacteriostatic formulations (Lopez-Lima et al., 2021). Its broad-spectrum fungicide action has made Cu essential, especially for organic agriculture where the management of fungal disease depends almost exclusively on its use (La Torre et al., 2018).

On the other hand, excessive use of Cu in agriculture and industry has significantly increased its concentration in cultivable soils. High levels of Cu inhibit plant development and growth due to irreversible morphological, physiological, and biochemical alterations (Mir et al., 2021). In fact, a complex and articulated system of utility, toxicity, absorption, and transport in plants has led to a dual outlook for Cu, as a harmful or an essential element (Shabbir et al., 2020). Predominantly present as a cupric ion (Cu^2+^), its bioavailability depends not only on the absolute concentration but also on the physicochemical characteristics of the soil. Organic and inorganic colloids immobilize Cu, making it unavailable to plants, especially in alkaline soils that represent approximately 30% of cultivable soils worldwide (Kumar et al., 2021; Wairich et al., 2022). In these cases, the foliar application of Cu is preferable to the soil application as it improves its own uptake and use efficiency, thus promoting a more sustainable agriculture. Although introduced to improve the nutritional status of animal organisms, synthetic chelating agents (EDTA and EDDHA) have become essential for the balanced nutrition of crop systems (Souri and Hatamian, 2019). However, in recent years, the unsustainability of the massive use of synthetic chelating agents in agriculture has emerged because the main artificial chelating agents used are persistent in natural systems because of their low biodegradability (Wu et al., 2004). In addition to this disadvantage, these compounds can alter the natural evolution of metals in groundwater and soil, making toxic metals bioavailable and compromising the quality of agricultural products and food safety (Zuluaga et al., 2023).

The necessary shift towards sustainable agricultural systems has highlighted the potential of innovative technical solutions, such as plant-based biostimulants. These latter can increase nutrient use efficiency and improve crop productivity and quality (Rouphael and Colla, 2020). The ability to produce biostimulants from plant waste has made vegetal protein hydrolysate (VPH) one of the most promising products (Xu and Geelen, 2018). Their biostimulant activity is attributable to a mixture of bioactive compounds such as amino acids, peptides, carbohydrates, minerals, phytohormones, phenols, and other organic compounds (Ciriello et al., 2022a). As Colla et al. (2015) stated, protein hydrolysates (PHs) can improve plant nutrition through three modalities: (i) promoting the growth of the root system, (ii) stimulating absorption processes, and (iii) increasing the availability of nutrients in the soil by increasing soil cation exchange and thus inorganic minerals’ solubility in water (Zuluaga et al., 2023). Combining biostimulants and innovative fertilizers such as chelates may represent a step forward in solving nutritional problems in agricultural systems. With their ability to act as ligand via non-covalent bonds, peptides may function as biochelators for specific microelements (Zuluaga et al., 2023). The use of biochelates makes micronutrients more available than standard fertilization practices (Souri et al., 2017). Moreover, the enhanced biodegradability of metal biochelates, compared to synthetic counterparts, provides a more favorable environmental impact. In the specific case of Cu, PHs might also provide additional benefits in terms of mitigation of metal-related stress in plants. Despite their potential, no in-depth studies have been conducted on the single and combined effects of biostimulants and the corresponding biochelates. The definition of synergism between Cu and PHs may offer novel solutions in the agronomic management of both plant nutrition and disease management. For this reason, our research aimed to compare the applications of copper sulfate pentahydrate, a VPH, Cu complexed with a vegetable protein hydrolysate (Cu-VPH), and a mixture of VPH and Cu-VPH on the yield and biochemical traits of Genovese basil grown in a glass greenhouse.

## 2 Materials and methods

### 2.1 Growth conditions, plant material, biostimulant application, and experimental design

The experimental trial was conducted on the experimental farm “Torre Lama” (lat. 43°31’N, long. 14°58’E; alt. 60 m above sea level) of the University of Naples “Federico II” - Department of Agriculture (DIA). The test was conducted in an unheated greenhouse with a galvanized iron structure and a glass roof. The structure has an aisle width of 10 m and a length of 30 m; the ridge height is 4.5 m, and the height at the eaves is 3.0 m. The plant material used for the trial was the *Ocimum basilicum* L. Genovese cultivar Mammolo (L’Ortolano, Cesena, Italy), grown in 1.2-L plots filled with agricultural soil at a density of 20 pt m^−2^, with 25 and 20 cm inter- and intra-rows, respectively. The main physical and chemical soil characteristics at the experimental site were clay loam texture (46% sand, 24.2% silt, and 29.8% clay), electrical conductivity (EC): 0.16 dS m^−1^, pH: 7.7, total nitrogen (N): 0.11%, and organic matter: 1.21% (*w*/*w*). The Olsen phosphorus and exchangeable potassium were 88 and 980 mg kg^−1^, respectively. The seeds were transplanted on 12 June 2019, in the two- to the three-leaf stage and irrigated using a micro-irrigation system with 16-mm inner diameter drip wings and a 10-cm pitch with self-compensating drippers of 2 L h^–1^ flow rate. The experimental trial involved randomized blocks with five treatments replicated three times. The experimental unit consisted of 15 plants. Seven days after transplanting (DAT), the crop was subjected to the following treatments: copper sulfate pentahydrate CuSO_4_-5H_2_O (0.23 g L^–1^; henceforth Cu), vegetal-protein hydrolysate at the manufacturer’s recommended dose (4 ml L^–1^ of Trainer^®^; henceforth VPH; Hello Nature, Rivoli Veronese, Italy), Cu complexed with peptides and amino acids of vegetal origin (Scudo^®^ - Hello Nature, Rivoli Veronese, Italy—at the dose of 0.5 ml L^–1^; henceforth Cu-VPH), Cu complexed with peptides and amino acids of vegetal origin (Scudo^®^) enriched with vegetal-protein hydrolysate Trainer^®^ (0.5 ml L^–1^ and 3.7 ml L^–1^, respectively; henceforth VPH+Cu-VPH), and untreated control. VPH was produced through enzymatic hydrolysis of legume biomass as reported by Colantoni et al. (2017) while the Cu complexed with peptides and amino acids (Cu-VPH) was obtained by chemical reaction of copper sulfate with peptides and amino acids contained in the VPH. Copper concentration in all Cu treatments (Cu, Cu-VPH, and VPH-Cu-VPH) was 58.5 mg L^−1^. In both VPH and VPH-Cu-VPH treatments, nitrogen as free amino acids and soluble peptides was 0.24 g L^−1^ while Cu-VPH treatment provided 0.02 g L^−1^ of N as amino acids and soluble peptides. Treatments were made by foliar application, always in the early morning, using separators to prevent drift contamination. By weekly applications, four treatments were made throughout the crop cycle.

At 37 DAT, eight plants per replicate were sampled to determine biometric parameters. Specifically, leaves and stems were separated to fresh weight (fw; g plant^–1^). The fresh yield was expressed as kg m^–2^. The sampled plant material was then placed in a ventilated oven (for 72 h at 65°C) to assess the total dry weight (dw) of leaves and stems and thus the dry matter (%).

### 2.2 Determination of CIELab colorimetric and SPAD indexes

Color coordinates (L, a*, and b*) were recorded using a Minolta Chroma Meter CR-300 handheld colorimeter (Minolta Camera Co. Ltd., Tokyo, Japan). Ten measurements were taken on the surface of fully expanded young leaves of eight plants per experimental unit on the day before harvest (36 DAT). The same leaves were used to determine the SPAD index using the SPAD-502 optical device instrument (Minolta Corp. Ltd., Osaka, Japan).

### 2.3 Determination of leaf gas exchange and chlorophyll fluorescence

At 37 DAT, between 10:30 a.m. and 1:30 p.m., in order to check the physiological status of plants at harvest, gas exchange and chlorophyll fluorescence measurements were made. Measurements were performed on fully expanded young basil leaves, avoiding the midrib. The maximum quantum efficiency of photosystem II (PSII) Fv/Fm was assessed on the leaves of nine plants per experimental unit after their adaptation to darkness (for at least 10 min) using leaf clips specific for the portable fluorometer Fv/Fm Meter (Opti-Sciences Inc., Hudson, USA). On the same leaves, the assimilation rate (A_CO2_) and transpiration rate (E) were determined using a portable gas exchange analyzer (LCA 4; ADC BioScientific Ltd., Hoddesdon, United Kingdom). The ratio between A_CO2_ and E was used to determine the instantaneous water use efficiency (WUEi). For leaf gas exchange measurements, all parameters [CO_2_ concentration, photosynthetically active radiation (PAR), and relative humidity (RH)] were set to ambient values while airflow was set to 400 ml s^–1^.

### 2.4 Determination of macro- and trace elements

The determination of macro- and trace elements was carried out as detailed by Ciriello et al. (2023). All standards and reagents used to determine macro- and trace elements by inductively coupled plasma optical emission spectrometric analysis (ICP OES) were purchased from Sigma-Aldrich (Milan, Italy). Calibration standards were prepared from the multi-element standard solution ICP Calibration mix EH61 PrimAg (Romil, Cambridge, UK). For accuracy determination, Standard Reference Material (SRM) 1570a (NIST) was prepared for trace elements in spinach (*Spinacia oleracea* L.) leaves, which was analyzed in triplicate in the same manner as the samples. All glassware and instruments were decontaminated by soaking them in a sulfuric acid solution overnight and then rinsed several times, first with deionized water and then with ultrapure water (Merck Millipore, Darmstadt, Germany).

The basil leaves were washed with ultrapure water and placed in a forced-ventilated oven at 65°C until they reached a constant weight and were finely ground. Before the analysis of the ICP OES (Spectroblue, Spectro Ametek, Berwyn, PA, USA), 0.5 g of the dried plant sample was weighed and subjected to laboratory analysis. Five grams of the dried plant sample, weighed in PTFE (polytetrafluoroethylene) vessels, was treated by microwave-assisted digestion (MLS-1200, Microwave Laboratory Systems, Milestone, Shelton, CT, USA) in a mixture of 9 ml of nitric acid (70%, for trace analysis), 3 ml of hydrochloric acid (30%, for trace analysis), and 0.5 ml of H_2_O_2_. The heating program consisted of switching from 20°C to 180°C in 15 min and then staying at 180°C for an additional 10 min, with a power setting of 800 W. The fully digested samples, after cooling, were transferred to a volumetric flask and the volume was increased to 50 ml with ultrapure water (Volpe et al., 2015).

Macro- and trace element analysis of the digested samples was performed by ICP OES. The plasma source was provided by 99.999% purity argon (SOL, Marcianise, Italy). The instrument was optimized daily for the maximum signal and tuned with a specific solution for the wavelength range studied. For non-alkali elements (Fe, Mn, Cu, Zn, and P), the calibration curve was constructed in the 1.0–100 mg L^–1^ range. For alkaline elements (K, Ca, and Mg), the calibration curve was constructed from 2 mg L^–1^ to 1,000 mg L^–1^. Macro (K, Ca, Mg, and P)- and trace elements (Cu, Fe, Mn, and Zn) were expressed as mg g^–1^ fw and µg g^–1^ fw, respectively, based on the original dw of each basil sample.

### 2.5 UHPLC-ESI/QTOF-MS untargeted metabolomics

Basil leaves were collected, ground with liquid nitrogen using pestle and mortar, and successively extracted and analyzed according to Rouphael et al. (2020). Briefly, a 1-g aliquot per sample was extracted in 15 ml of an aqueous solution with 80% (*v/v*) methanol acidified with 0.1% formic acid using an Ultra-Turrax (Polytron PT, City, Switzerland). Thereafter, the extracts were centrifugated at 8,000 × *g* for 10 min and 1 ml of each resulting supernatant was transferred into a glass vial by filtering through a 0.22-μm cellulose syringe filter for analysis.

Untargeted metabolomic analysis was performed using a 1290 ultrahigh-pressure liquid chromatographic system coupled to a G6550 electrospray quadrupole-time-of-flight mass spectrometry (UHPLC-ESI/QTOF-MS) from Agilent Technologies (Santa Clara, CA, USA). Reverse-phase chromatographic separation used an Agilent 120 PFP column (100 mm × 2.1 i.d., 1.9 μm particle size) and a mobile phase linear gradient of water and acetonitrile (6-94% acetonitrile in 33 min). Both mobile phases were acidified with 0.1% (*v/v*) formic acid. The mass spectrometer operated in full scan mode (range 100–1200 m/z), positive polarity (ESI+), and source parameters were previously optimized (Mastinu et al., 2021).

Compound annotation was achieved with MussHunter Profinder 10.0 software (Agilent Technologies) by applying the “find-by-formula” algorithm, based on the combination of monoisotopic accurate mass (tolerance of 5 ppm) and isotopic spacing and ratio, following mass and retention time alignment. The PlantCyc 9.6 [http://www.plantcyc.org/; Hawkins et al. (2021)] database was used for annotation, and post-annotation filtering retained the compounds present in three replications within at least one treatment. According to COSMOS Metabolomics Standard Initiative, Level 2 of annotation, corresponding to putatively annotated compounds, was adopted (Salek et al., 2015).

### 2.6 Statistics

All data were analyzed with IBM SPSS Statistics software version 26.0 (SPSS Inc., Chicago, IL, USA) and are presented as mean ± standard error, *n* = 3. All mean effects were subjected to one-way ANOVA analysis. Statistical significance was determined with Tukey’s HSD test at the *p* = 0.05 level.

Mass Profiler Professional 15.1 software (Agilent Technologies) was used to elaborate metabolomic data. Compound abundance was Log2 transformed and normalized at the 75th percentile, then baselined against the median. Thereafter, the unsupervised hierarchical cluster analysis (Ward linkage method and Euclidean distance) was carried out to get an overview of phytochemical patterns and investigate relatedness among treatments. Then, the raw dataset was imported into SIMCA 13 (Umetrics, Malmo, Sweden), pareto scaled, and subjected to the supervised orthogonal projection to latent structures discriminant analysis (OPLS-DA). The OPLS-DA model was cross- validated (CV-ANOVA) and R2Y and Q2Y parameters (degree of correlation and prediction ability, respectively) were recorded. Moreover, overfitting was excluded through a permutation test (*n* = 100), outliers were inspected through Hotelling’s T2, and discriminant compounds selected by variable importance in projection (VIP) in the predictive model were noted. Finally, Volcano plot analysis (*p*-value < 0.05, Benjamini correction; fold change ≥ 2) was used to identify differential compounds, and the PlantCyc pathway tools software [http://www.plantcyc.org/; Caspi et al. (2014)] was used for biochemical interpretations.

## 3 Results

### 3.1 Yield and yield parameters

With the exception of dry matter, the yield and yield components presented in **Table 1** showed significant differences among the treatments (Control, Cu, Cu-VPH, VPH+Cu-VPH, and VPH). Specifically, when compared to the Control group, the VPH+Cu-VPH treatment exhibited notable increases in leaf fresh weight, total fresh weight, stem dry weight, total dry weight, and fresh yield, with improvements of 16.80%, 18.62%, 29.50%, 22.70%, and 18.48%, respectively.

**Table 1:** Leaf, stem, and total fresh weight; leaf, stem, and total dry weight; fresh yield; and dry matter of basil under untreated control, copper sulfate (Cu), copper complexed with amino acids and peptides (Cu-VPH), vegetal protein hydrolysate (VPH), and Cu-VPH enriched with VPH (VPH+Cu-VPH) treatments.

Treatments	Leaf fresh weight	Stem fresh weight	Total fresh weight	Leaf dry weight	Stem dry weight	Total dry weight	Fresh yield	Dry matter
	g plant^–1^						kg m^–2^	%
Control	62.56 ± 0.23 c	29.39 ± 0.42 b	91.95 ± 0.53 c	6.97 ± 0.07 b	3.39 ± 0.04 d	10.35 ± 0.08 c	1.84 ± 0.01 c	11.26 ± 0.03
Cu	64.22 ± 0.80 c	31.25 ± 0.93 b	95.47 ± 1.67 c	6.94 ± 0.06 b	3.87 ± 0.07 bc	10.80 ± 0.12 bc	1.91 ± 0.03 c	11.32 ± 0.12
Cu-VPH	66.38 ± 0.52 b	34.47 ± 0.73 a	100.85 ± 1.25 b	7.42 ± 0.14 b	3.71 ± 0.08 c	11.14 ± 0.19 bc	2.02 ± 0.03 b	11.04 ± 0.06
VPH+Cu-VPH	73.07 ± 0.14 a	36.00 ± 0.43 a	109.07 ± 0.57 a	8.32 ± 0.26 a	4.39 ± 0.07 a	12.70 ± 0.32 a	2.18 ± 0.01 a	11.64 ± 0.24
VPH	67.52 ± 0.27 b	34.69 ± 0.28 a	102.21 ± 0.37 b	7.67 ± 0.19 ab	4.00 ± 0.02 b	11.68 ± 0.19 b	2.04 ± 0.01 b	11.42 ± 0.15
Significance	***	***	***	***	***	***	***	ns

ns and *** denote nonsignificant or significant at *p* ≤ 0.001, respectively. Different letters within each column indicate significant differences according to Tukey HSD test (*p* = 0.05). All data are expressed as mean ± standard error, n = 3.

Relative to stem fresh weight, the highest values were obtained from the Cu-VPH, VPH+Cu-VPH, and VPH treatments, with no significant differences. Compared with the Cu-VPH treatment, the combined VPH+Cu-VPH treatment significantly increased leaf dry weight (+12.13%).

### 3.2 Physiological parameters

All physiological parameters, shown in **Table 2**, were significantly affected by the treatments under investigation. The highest values of net CO_2_ assimilation rate (A_CO2_; 14.38 μmol CO_2_ m^–2^ s^–1^) and fluorescence (0.81) were obtained in plants of the VPH+Cu-VPH treatment. In comparison with the Control, the Cu-VPH and VPH+Cu-VPH treatments increased transpiration (E) by 49.10% (on average). In contrast, the Cu-VPH treatment reduced WUEi (–29.91%) compared with the Control.

**Table 2:** Net CO_2_ assimilation rate (A_CO2_), transpiration (E), water use efficiency (WUE), and chlorophyll fluorescence (Fv/Fm) of basil under untreated control, copper sulfate (Cu), copper complexed with amino acids and peptides (Cu-VPH), vegetal protein hydrolysate (VPH), and Cu-VPH enriched with VPH (VPH+Cu-VPH) treatments.

Treatments	A_CO2_	E	WUEi	Fluorescence Fv/Fm
	μmol CO_2_ m^−2^ s^−1^	mol H_2_O m^−2^ s^−1^	μmol CO_2_ mol^−1^ H_2_O	
Control	11.15 ± 0.11 b	1.66 ± 0.04 c	6.72 ± 0.12 a	0.76 ± 0.00 d
Cu	11.96 ± 0.13 b	2.18 ± 0.18 b	5.57 ± 0.45 bc	0.77 ± 0.00 c
Cu-VPH	12.19 ± 0.13 b	2.59 ± 0.04 a	4.71 ± 0.10 c	0.78 ± 0.00 b
VPH+Cu-VPH	14.38 ± 0.45 a	2.36 ± 0.03 ab	6.09 ± 0.12 ab	0.81 ± 0.00 a
VPH	12.14 ± 0.19 b	2.00 ± 0.02 bc	6.07 ± 0.15 ab	0.77 ± 0.00 c
Significance	***	***	***	***

*** significant at *p* ≤ 0.001. Different letters within each column indicate significant differences according to Tukey HSD test (*p* = 0.05). All data are expressed as mean ± standard error, n = 3.

### 3.3 Protein, carbohydrate, and polyphenols concentration

Cu-VPH and VPH biostimulant application significantly affected soluble protein content. The protein content was significantly decreased by applying Cu-VPH and VPH, which was 25.6% and 31.6% lower than in untreated basil plants, respectively (**Table 3**). None of the treatments significantly affected starch (avg. 144.8 µmol g^–1^ dw) and sucrose (2.3 µmol g^–1^ dw) concentrations. (**Table 3**). However, the application of VPH+Cu-VPH averaged over other treatments positively affected the glucose concentrations in basil leaves (an increase of 76.2% compared to control), even if also Cu and Cu-VPH determined an increase of this soluble sugar of approximately 40% (**Table 3**). Fructose increased 24.4% and 30.8% compared to control when treated with Cu-VPH and VPH+Cu-VPH, respectively (**Table 4**). Finally, Cu-VPH and VPH significantly decreased the polyphenols content, with no significant differences among the three treatments (**Table 3**).

**Table 3:** Soluble proteins, glucose, fructose, sucrose, starch, and polyphenols concentration of basil under untreated control, copper sulfate (Cu), copper complexed with amino acids and peptides (Cu-VPH), vegetal protein hydrolysate (VPH), and Cu-VPH enriched with VPH (VPH+Cu-VPH) treatments.

Treatments	Soluble proteins	Glucose	Fructose	Sucrose	Starch	Polyphenols
	mg g^–1^ fw	µmol g^–1^ dw				mg GAE g^–1^ dw
Control	9.11 ± 0.59 a	36.96 ± 1.41 c	22.90 ± 2.87 b	2.65 ± 0.50	146.27 ± 10.43	20.21 ± 0.22 a
Cu	7.85 ± 0.34 ab	51.65 ± 2.33 b	23.17 ± 1.12 b	2.76 ± 0.30	152.57 ± 24.54	18.92 ± 0.91 a
Cu-VPH	6.78 ± 0.47 b	51.23 ± 0.27 b	28.50 ± 0.89 a	2.10 ± 0.31	148.91 ± 25.37	16.40 ± 0.48 b
VPH+Cu-VPH	7.26 ± 0.33 ab	65.12 ± 3.75 a	29.96 ± 0.50 a	2.37 ± 0.28	160.45 ± 3.04	15.79 ± 0.42 b
VPH	6.23 ± 0.14 b	44.88 ± 0.89 bc	27.40 ± 1.58 ab	1.68 ± 0.20	115.66 ± 19.12	15.38 ± 0.36 b
Significance	**	***	*	ns	ns	***

ns, *, **, and *** denote nonsignificant or significant at *p* ≤ 0.05, 0.01, and 0.001, respectively. Different letters within each column indicate significant differences according to Tukey HSD test (*p* = 0.05). All data are expressed as mean ± standard error, n = 3.

**Table 4:** Aminogram, essential amino acids, branched-chain amino acid (BCAAs), and total amino acids of basil under untreated control, copper sulfate (Cu), copper complexed with amino acids and peptides (Cu-VPH), vegetal protein hydrolysate (VPH), and Cu-VPH enriched with VPH (VPH+Cu-VPH) treatments.

Treatments	Control	Cu	Cu-VPH	VPH+ Cu-VPH	VPH	Significance
Ala	3.64 ± 0.34 b	5.41 ± 0.10 b	2.81 ± 0.39 b	9.68 ± 1.63 a	5.93 ± 0.73 ab	***
Arg	0.908 ± 0.13 b	1.771 ± 0.08 a	1.436 ± 0.11 ab	0.959 ± 0.13 b	1.757 ± 0.22 a	**
Asn	1.142 ± 0.226 b	0.970 ± 0.087 b	1.061 ± 0.087 b	2.634 ± 0.486 a	1.627 ± 0.204 ab	**
Asp	0.513 ± 0.023 b	0.374 ± 0.041 b	0.410 ± 0.047 b	0.957 ± 0.137 a	1.018 ± 0.106 a	***
GABA	1.825 ± 0.367 ab	2.666 ± 0.339 a	0.700 ± 0.126 b	2.816 ± 0.439 a	2.206 ± 0.374 ab	**
Gln	2.028 ± 0.273 ab	1.356 ± 0.099 b	1.209 ± 0.234 b	3.619 ± 0.594 a	3.805 ± 0.532 a	**
Glu	18.49 ± 1.17 ab	16.43 ± 1.85 ab	10.55 ± 0.68 b	20.15 ± 2.78 a	20.49 ± 2.06 a	*
Gly	0.511 ± 0.068 abc	0.443 ± 0.024 bc	0.275 ± 0.044 c	0.714 ± 0.055 a	0.685 ± 0.067 ab	***
His	0.040 ± 0.006 b	0.165 ± 0.018 b	0.136 ± 0.014 b	0.369 ± 0.052 a	0.357 ± 0.040 a	***
Ile	0.781 ± 0.099 ab	0.896 ± 0.048 ab	0.543 ± 0.036 c	1.155 ± 0.090 a	1.006 ± 0.125 a	**
Leu	1.454 ± 0.130 ab	1.465 ± 0.082 ab	1.088 ± 0.096 b	1.850 ± 0.094 a	1.641 ± 0.186 ab	*
Lys	2.454 ± 0.068	2.411 ± 0.097	2.176 ± 0.221	2.318 ± 0.146	2.464 ± 0.057	ns
MEA	0.919 ± 0.156 a	0.773 ± 0.052 ab	0.299 ± 0.034 b	1.207 ± 0.183 a	1.149 ± 0.163 a	**
Met	0.028 ± 0.003 ab	0.033 ± 0.006 a	0.015 ± 0.002 b	0.031 ± 0.003 ab	0.031 ± 0.004 ab	*
Orn	1.108 ± 0.098	1.217 ± 0.125	0.904 ± 0.070	1.313 ± 0.099	1.214 ± 0.127	ns
Phe	0.201 ± 0.028 bc	0.238 ± 0.004 bc	0.130 ± 0.013 c	0.442 ± 0.044 a	0.322 ± 0.066 ab	**
Pro	2.584 ± 0.158	2.051 ± 0.055	2.261 ± 0.215	2.332 ± 0.107	2.067 ± 0.304	ns
Ser	0.611 ± 0.048 bc	0.517 ± 0.049 bc	0.394 ± 0.048 c	0.897 ± 0.037 a	0.634 ± 0.055 b	***
Thr	0.382 ± 0.054 ab	0.250 ± 0.021 b	0.166 ± 0.028 b	0.632 ± 0.095 a	0.465 ± 0.092 ab	**
Trp	0.403 ± 0.083 ab	0.495 ± 0.002 ab	0.251 ± 0.026 b	0.752 ± 0.116 a	0.530 ± 0.101 ab	*
Tyr	0.354 ± 0.063 ab	0.338 ± 0.009 ab	0.218 ± 0.018 b	0.518 ± 0.057 a	0.407 ± 0.076 ab	*
Val	0.850 ± 0.174 ab	1.044 ± 0.057 ab	0.743 ± 0.038 b	1.471 ± 0.128 a	1.231 ± 0.204 ab	*
Essential AA	7.50 ± 0.74 bc	8.77 ± 0.28 abc	6.68 ± 0.54 c	9.98 ± 0.65 ab	10.14 ± 0.44 a	**
BCAAs	3.08 ± 0.40 ab	3.40 ± 0.19 ab	2.37 ± 0.17 b	4.48 ± 0.29 a	3.88 ± 0.51 ab	*
Total AA	51.81 ± 4.34 bc	53.49 ± 2.12 ab	36.83 ± 2.72 c	67.94 ± 4.29 a	68.05 ± 3.02 a	***

ns, *, **, and *** denote nonsignificant or significant at *p* ≤ 0.05, 0.01, and 0.001, respectively. Different letters within each row indicate significant differences according to Tukey HSD test (*p* = 0.05). All data are expressed as mean ± standard error, n = 3. All data were expressed as µmol g^–1^ dw.

### 3.4 Free amino acid concentration

The free amino acid profile of basil plants as a function of the different treatments is reported in **Table 4**. Irrespective of the treatments, glutamate and alanine were quantitatively the major amino acids, representing, on average, approximately 31.0% and 9.9% of total free amino acids in basil plants, respectively. Essential amino acids (the sum of isoleucine, histidine, leucine, lysine, methionine, threonine, and valine) accounted, on average, for 15.5% of total amino acids. Interestingly, the VPH and VPH+Cu-VPH determined a significant increase of all amino acids, particularly glutamate, glutamine, aspartate, and asparagine, which are involved in nitrogen assimilation and transport (**Table 4**). In contrast, the application of Cu-VPH alone determined the opposite effect, with a strong decrease of total amino acid content and of GABA (–61.6%), MEA (–67.5%), phenylalanine (–35.3%), glutamate (–42.9%), and alanine (–22.8%); even the latter two were not significant (**Table 4**). In this condition, the only amino acids that did not decrease compared to the respective controls were the amides asparagine and glutamine, proline, and essential amino acids (**Table 4**).

### 3.5 Leaf mineral composition

Among the analyzed minerals, Mn and K were by far the most abundant micro- and macroelements. Regardless of the treatments, K values ranged from 52.1 to 59.7 mg g^–1^ dw, while Mn values ranged from 56.5 to 74.2 µg g^–1^ dw (**Table 5**). None of the treatments affected the concentration of P (averaging at 2.4 mg g^–1^ dw). However, the Cu, Cu-VPH, and VPH+Cu-VPH treatments significantly increased the Cu concentration by 15.3-, 15.0-, and 7.8-fold, respectively, compared to the control (**Table 5**). The Cu-VPH treatment also significantly increased K (+14.3%), Mg (+18.0%), and Fe (+29.2%), while the VPH+Cu-VPH treatment significantly increased Ca (+24.7%) and Mn (+20.8%) compared to their respective control treatments. The VPH treatment significantly reduced Zn by 21.93% compared to the VPH+Cu-VPH treatment (**Table 5**).

**Table 5:** Mineral profile of basil leaves under untreated control, copper sulfate (Cu), copper complexed with amino acids and peptides (Cu-VPH), vegetal protein hydrolysate (VPH), and Cu-VPH enriched with VPH (VPH+Cu-VPH) treatments.

Treatments	P	K	Ca	Mg	Cu	Fe	Mn	Zn
	mg g^–1^ dw				μg g^–1^ dw			
Control	2.61 ± 0.11	52.10 ± 1.05 b	16.54 ± 0.62 b	3.20 ± 0.06 c	7.78 ± 0.65 c	34.06 ± 1.18 b	61.37 ± 0.54 bc	19.19 ± 0.59 ab
Cu	2.41 ± 0.12	53.02 ± 1.29 b	16.76 ± 0.48 b	3.30 ± 0.12 c	118.66 ± 6.81 a	33.41 ± 1.58 b	66.10 ± 2.23 b	18.40 ± 0.55 b
Cu-VPH	2.42 ± 0.14	59.59 ± 1.56 a	18.65 ± 0.58 ab	3.78 ± 0.05 ab	116.87 ± 8.78 a	44.01 ± 1.98 a	65.80 ± 1.99 b	18.76 ± 0.74 ab
VPH+Cu-VPH	2.36 ± 0.01	57.73 ± 1.39 ab	20.63 ± 0.98 a	3.94 ± 0.13 a	60.66 ± 2.53 b	44.83 ± 2.76 a	74.17 ± 0.87 a	21.56 ± 0.77 a
VPH	2.32 ± 0.07	59.72 ± 0.93 a	17.17 ± 0.16 b	3.50 ± 0.08 bc	6.98 ± 0.64 c	40.32 ± 1.20 ab	56.52 ± 0.66 c	16.83 ± 0.50 b
Significance	ns	**	**	***	***	**	***	**

ns, **, and *** denote nonsignificant or significant at p ≤ 0.01 and 0.001, respectively. Different letters within each column indicate significant differences according to Tukey HSD test (p = 0.05). All data are expressed as mean ± standard error, n = 3.

### 3.6 Principal component analysis

To obtain an in-depth overview of growth parameters, mineral profile, and nutritional and functional quality traits of the studied basil leaves in response to the diverse treatments, a principal component analysis (PCA) was carried out for all the analyzed parameters discussed above. The first three principal components (PCs) were related with eigenvalues higher than 1 and explained 92.6% of the total variance, with PC1, PC2, and PC3 accounting for 54.2%, 25.1%, and 13.3%, respectively (data not shown).

**Figure 1:**
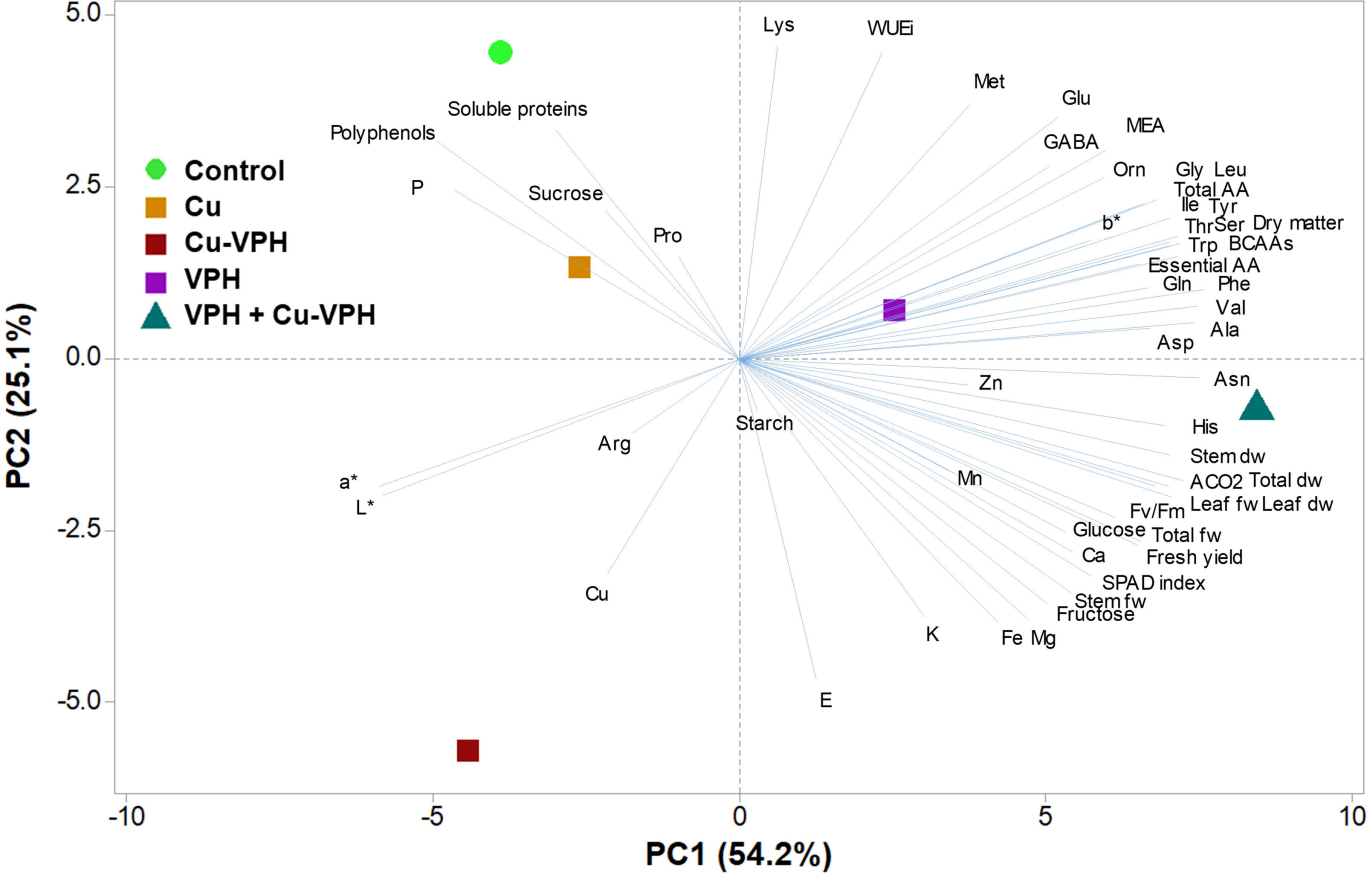
Principal component loading plot and scores of principal component analysis (PCA) of growth parameters, mineral elements, SPAD index, starch, soluble carbohydrates, polyphenols, and total amino acids in basil leaves untreated (Control) or foliarly treated with copper sulfate (Cu), copper complexed with amino acids and peptides (Cu-VPH), vegetal protein hydrolysate (VPH), and Cu-VPH enriched with VPH (VPH+Cu-VPH).

The increase in metabolism reshaping (activation of more metabolic pathways) contributed to the clear separation of PC1 with VPH+Cu-VPH in the positive side of PC1. In contrast, the distribution on PC2 was more linked to the efficient use of water with the control and Cu-VPH in the positive and negative side of PC2, respectively (**Figure 1**). PC1 was positively correlated to amino acids [in particular phenylalanine, asparagine, valine, tryptophan, branched-chain amino acids (BCAAs), tyrosine, threonine, and serine], dry matter, leaf fresh weight, stem dry weight, leaf dry weight, total dry weight, and A_CO2_ (**Figure 1**). PC1 negatively correlated with P, polyphenols, and L* and a* parameters. PC2 was positively correlated with lysine and WUEi, and negatively correlated with E (**Figure 1**). In addition, basil plants treated with VPH+Cu-VPH positioned on the positive side of PC1 close to the *x*-axis between the lower and upper right quadrant produced leaves of premium quality with higher fresh yield and concentrations of free amino acids and, in particular, essential amino acids. Control and Cu-treated plants in the upper left quadrant were instead characterized by the lowest fresh yield but the highest polyphenols content. Finally, Cu-VPH showed the lowest amino acid content, lower soluble proteins, and polyphenols than the control, but a high mineral concentration (K, Mg, Cu, and Fe) (**Figure 1**).

### 3.7 UHPLC-ESI/QTOF-MS metabolomic profile of basil

UHPLC-ESI/QTOF-MS untargeted metabolomics was carried out to understand the effect of the different treatments on the basil metabolome. Overall, more than 3,200 leaf compounds were putatively annotated and provided as **Supplementary Material**, together with individual abundances and composite mass spectra (**Supplementary File 1**).

**Figure 2:**
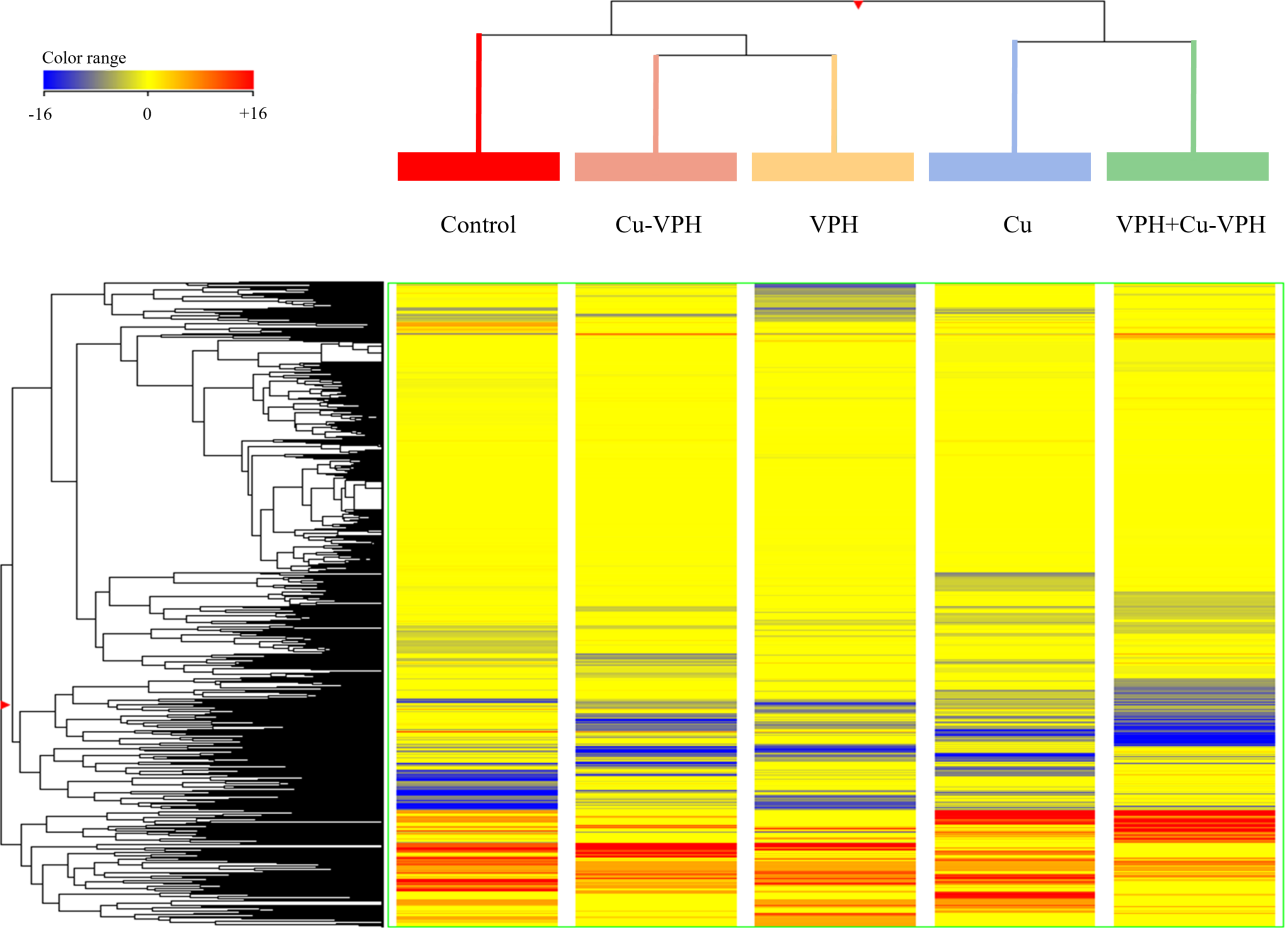
Fold-change heatmap obtained from the unsupervised hierarchical cluster analysis (Euclidean distance; linkage rule: Ward) on UHPLC-ESI/QTOF-MS data of basil leaf chemical profiles under untreated control, copper sulfate (Cu), copper complexed with amino acids and peptides (Cu-VPH), vegetal protein hydrolysate (VPH), and Cu-VPH enriched with VPH (VPH+Cu-VPH) treatments.

Based on these metabolomic signatures, unsupervised hierarchical clustering was first performed to describe similarities/dissimilarities among the treatments. As shown in **Figure 2**, two main clusters were generated by the fold-change-based heatmap, one represented by Cu and VPH+Cu-VPH, and another by control, Cu-VPH, and VPH. Within this last one, a subcluster—containing Cu-VPH- and VPH-treated samples—was created, indicating a more comparable metabolic profile in the basil plant after the two treatments.

**Figure 3:**
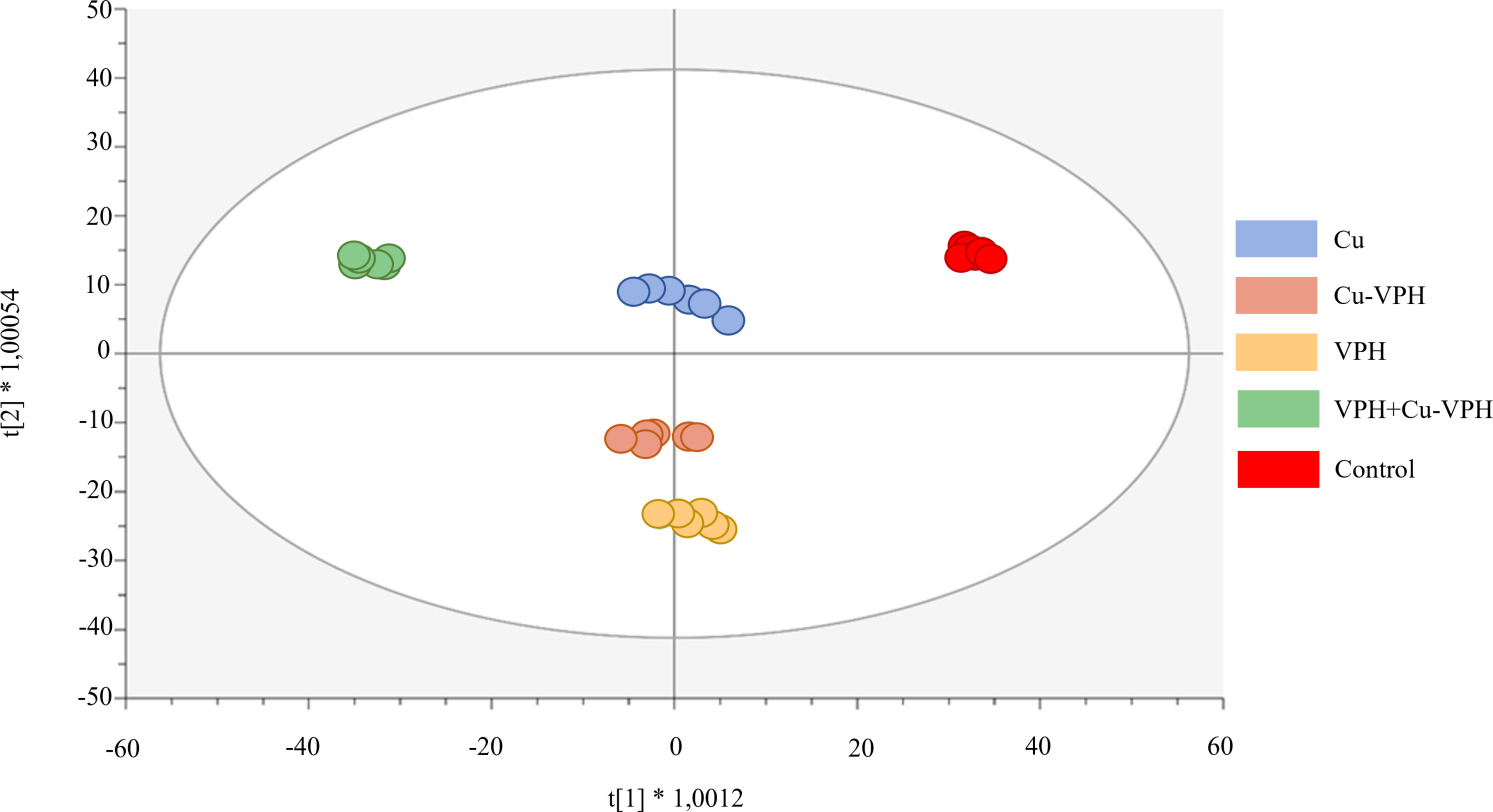
Orthogonal Projections to Latent Structures Discriminant Analysis (OPLS-DA) score plot for basil leaf under untreated control, copper sulfate (Cu), copper complexed with amino acids and peptides (Cu-VPH), vegetal protein hydrolysate (VPH), and Cu-VPH enriched with VPH (VPH+Cu-VPH) treatments.

Successively, the supervised OPLS-DA multivariate analysis allowed us to better outline the differences between treatments and to highlight discriminant (VIP) compounds underlying these differences. The OPLS-DA score plot, provided in **Figure 3**, revealed distinct phytochemical profiles in basil leaves after treatments. In particular, the first latent vector discriminated VPH+Cu-VPH from the control, with the treated replicates being considerably separated from the untreated ones. This result indicated the more pronounced effect of VPH+Cu-VPH on metabolic reprogramming of basil leaves, compared to the other treatments. Differently, the second latent vector pointed out a differential profile of VPH from Cu-VPH and control. The OPLS model was validated through CV-ANOVA (*p*-value = 1.87E-15), and adequate accuracy parameters (R2Y = 0.99 and Q2Y = 0.88) were obtained.

**Figure 4:**
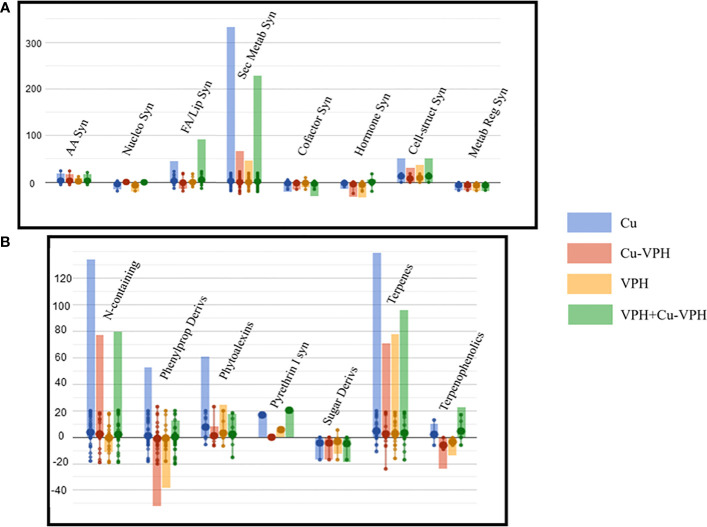
Leaf basil metabolic processes **(A)** and the relative details of secondary metabolism **(B)** as affected by copper sulfate (Cu), copper complexed with amino acids and peptides (Cu-VPH), vegetal protein hydrolysate (VPH), and Cu-VPH enriched with VPH (VPH+Cu-VPH) treatments compared to untreated control. The compounds resulting from Volcano plot analysis (*p* value < 0.05; fold change > 0.01) were loaded into the PlantCyc Pathway Tool (https://www.plantcyc.org/). The *x*-axis represents each set of metabolic subcategories, while the *y*-axis corresponds to the cumulative log fold change (FC). The large dots represent the average (mean) of all FCs for the different metabolites in the class, while the small dots represent the individual log FC.

The list of VIP compounds, which mostly discriminated the treatments (VIP > 1.5), is provided in **Supplementary File 1**. Overall, isoprenoids were the most represented class of metabolites, mainly including carotenoids, sesquiterpenes, and gibberellins. Finally, Volcano plot analysis (*p*-value < 0.05; fold change > 2) was carried out to select those compounds involved in basil plants’ response to the treatments. A total of 251 differential metabolites were identified and provided in **Table S3** together with their function class. Therein, compounds related to secondary metabolite biosynthesis were the most represented in all treatments, followed by fatty acid and lipid biosynthesis-related metabolites. The graphical interpretation obtained with the PlantCyc pathway tool analysis allowed us to get insight into the metabolic pathways mostly affected in basil leaves (**Figure 4**).

Notably, secondary metabolism was strongly modulated, showing an overall upregulation after each treatment. Among secondary metabolites, terpene and nitrogen-containing compounds were markedly modulated, followed by phenylpropanoid derivates. Notably, Cu elicited the highest accumulation of metabolites of each of these classes, reporting above all higher concentration of alkaloids [(S)-laudanosine, (S)-N-methylcoclaurine, 16-hydroxytabersonine, and galanthamine] and terpenoids, including carotenoids (lactucaxanthin, β-citraurin, and 9′-cis-neoxanthin), diterpenoids, and sesquiterpenoids (desoxyhemigossypol and 2-dehydrolubimin). Interestingly, increased levels of terpenoid phytoalexins (e.g., momilactone A and 2-dehydrolubimin) were noticed in Cu-treated samples. Nevertheless, an up-accumulation for both these two classes was observed with the other treatments, except for VPH, which exhibited a slight decrease of alkaloid and glucosinolate compounds (2-benzoyloxy-3-butenylglucosinolate and 2-sinapoyloxy-3-butenylglucosinolate).

Differently, improved contents of phenylpropanoids were highlighted only after VPH and Cu treatments. However, the strongest modulation was shown with Cu, highlighting particularly enhanced concentrations of (2E,4Z,6E)-5-formyl-2-hydroxy-8-oxoocta-2,4,6-trienoate, (+)-sesaminol, apigeninidin, and (+)-6a-hydroxymaackiain. In contrast, Cu-VPH and VPH showed a differential pattern, showing a general downregulation of phenylpropanoid compounds. Following both treatments, decreased levels of 8-C-glucosyl chrysin, rutin, (+)-dihydrokaempferol, and diphyllin were observed.

## 4 Discussion

Remarkable changes in morpho-physiological and biochemical traits were observed under VPH+Cu-VPH treatments compared to other ones. VPH+Cu-VPH treatment showed the highest leaf and total fresh and dry weight that increased the basil fresh yield. Probably, it was due to its higher carboxylation activity (A_CO2_) and maximum potential quantum efficiency of Photosystem II (Fv/Fm) compared to all other treatments. It may be supposed that the VPH+Cu-VPH treatment improved the Cu use efficiency, as well as the Ca and Mn ones. In particular, it enhanced Cu and Fe integration as cofactors in thylakoid electron transporters like plastocyanin and Fe-proteins (e.g., cytochromes, Fe-S clusters, and ferredoxin), and that of Ca and Mn in the water splitting system of the photosynthetic electron transport chain, which became capable of accommodating faster and higher rates of electron flow under high light (Printz et al., 2016). Also, photochemical quenching saturated at higher irradiance in these Cu-sufficient plants, thus increasing the effective quantum yield of photosynthesis compared to other treatments, particularly to control (Kim and Price, 2017). Instead, in the other treatments, particularly in control, the absorbed light energy might exceed the photosynthetic capacity, and over-reduce the photosynthetic electron transport chain, thus leading to the generation of reactive oxygen species (ROS) (Sperdouli et al., 2021). Accordingly, in control, the highest levels of polyphenols were found, since they are a powerful antioxidant particularly efficient in scavenging ROS and counteracting oxidative stress damage (Caldwell et al., 1983). Nevertheless, considering that treatments with higher levels of phenols are also less productive, it is probable that both the control plants and the Cu-treated plants have bolstered secondary metabolic pathways at the expense of primary ones (Prinsloo and Nogemane, 2018; Ciriello et al., 2022b). Phenolic compounds have an antioxidant activity higher than vitamins since they can prevent ROS formation and improve antioxidant cell response due to their ability to chelate metal ions (Faraloni et al., 2021). The problem of excess light correlated to high polyphenols content was not present in the other plant treatments, which showed higher transpiration and carboxylation activity, but significantly lower polyphenols, except for Cu treatment, even if at a lower extent, but without negative consequences on plant fresh weight. Interestingly, VPH+Cu-VPH-, VPH-, and Cu-VPH-treated plants also showed a high SPAD index accompanied by the deepest coloration resulting from more intense yellowness (b* values; **Supplementary Table S1**) and the highest content of glucose and free amino acids. In particular, in VPH+Cu-VPH plants, the increase in the amounts of leaf glutamate, aspartate, and amides (glutamine and asparagine) evidenced not only a higher N use efficiency but also (i) a higher energy availability, probably deriving by the more efficient photosynthesis, and by the capacity to store nitrogen in glutamine and asparagine instead of soluble proteins, with a much lower energy expense (Diab and Limami, 2016), and (ii) the possibility to use these two amides as long-distance nitrogen transport molecules, given their high N/C ratio, to supply organic nitrogen to roots to be used as amine donor for the synthesis of all protein amino acids boosting root growth (Van Oosten et al., 2019).

In contrast, the effect of Cu-VPH alone deserves particular attention. It determined the highest transpiration and the lowest WUEi and free amino acid levels, maintaining a high SPAD index, A_CO2_, Fv/Fm, mineral nutrients, and fresh yield. Noteworthy, among amino acids, GABA and MEA strongly decreased. It seems that GABA, which has a well-known antioxidant activity against ROS and is useful to stabilize and protect membranes and macromolecules, was not needed under Cu-VPH treatment (Carillo, 2018). This is also confirmed by the low levels of monoethanolamine (MEA), a serine derivative important for synthesizing or regenerating phospholipids (Carillo et al., 2020). This suggests that under Cu-VPH treatment, the higher Cu concentration, probably not yet toxic as in Cu treatment, can modulate plant metabolism in a way that photo-assimilates are promptly diverted to roots. In roots, N is mainly used to boost root growth and soil exploration, instead of remaining in leaves and being invested in proteins, particularly RUBISCO proteins that make up a large fraction of N in leaves. The higher amounts of water and mineral nutrients acquired exert a positive effect on plant metabolism, positively affecting processes such as chloroplast and mitochondrial electron transports, ROS detoxification, and redox status. However, since the high availability of water, Cu-VPH plants less strictly control transpiration, but without being able to further increase carboxylation activity; consequently, the lower A_CO2_-to-transpiration (E) ratio determines lower WUEi values compared to that of VPH+Cu-VPH (Liu et al., 2018).

**Figure 5:**
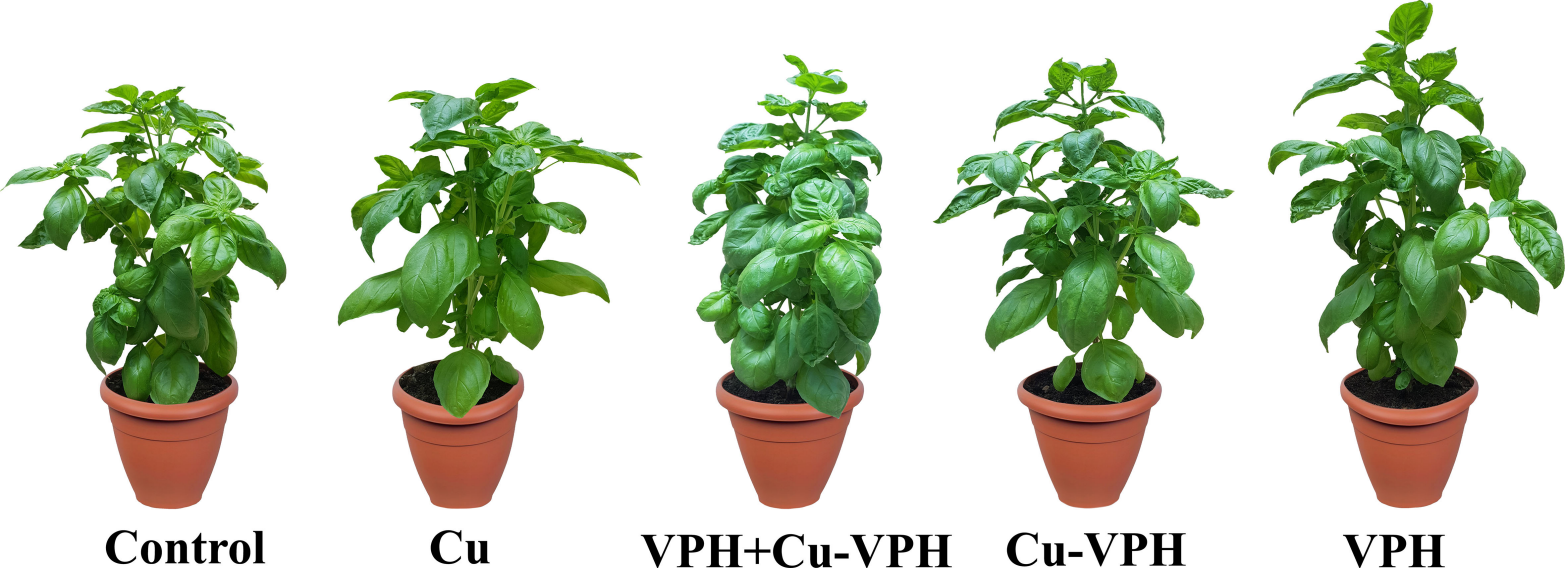
Illustrative picture of basil plants untreated (Control) or foliarly treated with copper sulfate (Cu), copper complexed with amino acids and peptides (Cu-VPH), vegetal protein hydrolysate (VPH), and Cu-VPH enriched with VPH (VPH+Cu-VPH).

VPH+Cu-VPH treatment increased, even if not significantly compared to the VPH one, the tryptophan content of plants, and determined an inhibition of stem elongation and an increase of compactness of plants, with a more equilibrated leaf enlargement (especially that of older leaves) and consequent increase of yield compared to all other differently treated plants (**Figure 5**).

This is puzzling since Colla et al. (2014) demonstrated that VPH (Trainer^®^) was able to induce coleoptile elongation in detached corn (*Zea mays* L.) seedlings due to the high tryptophan content equal to 3 g kg^–1^ that exerted an auxin-like activity (Rouphael et al., 2018). However, tryptophan, which is the main precursor of indole-3-acetic acid (IAA), not only can modulate shoot length, but also, in synergy with the peptides contained in VPH, can certainly contribute via auxin- and/or gibberellin-like activity, as shown in *in vitro* bioassays by Ertani et al. (2009) and Colla et al. (2014), to modulate/improve root architecture (Fukaki and Tasaka, 2009). This reshaping of root architecture has been correlated with an enhancement of N uptake and translocation known as “nutrient acquisition response” and can also be associated with the higher root assimilation of Ca, Mg, Mn, and Fe, and certainly also Cu, and their translocation to shoots of basil plants, as previously seen in Rouphael et al. (2017) and Carillo et al. (2019). This higher availability of N and other minerals can be responsible for the better leaf nutritional status and yield of VPH+Cu-VPH treatment. In particular, the higher availability of amino acids and Mg can contribute to the higher photochemical efficiency and activity of photosystem II (PSII), possibly due to the increase of photosynthetic proteins, particularly Ribulose-1,5-bisphosphate carboxylase/oxygenase (RuBisCO), and their Mg-dependent activation, thus increasing CO_2_ assimilation (A_CO2_) and translocation of recent photosynthates via the phloem to sink tissues (Carillo et al., 2019). Therefore, the interaction between VPH and Cu-VPH exerted a positive effect on the electron transport chain, CO_2_ carboxylation, and translocation of newly synthetized soluble sugars to young leaves, decreasing the photooxidative damage, the consequent ROS production, and the need to synthesize polyphenols, while increasing N use efficiency, amino acid synthesis, and growth. This phenomenon was phenotypically exteriorized by the change in plant shape and higher crop yield. Moreover, when Cu is supplied by foliar spraying, high contents of free cytosolic Cu^+^ in leaves may cause a massive generation of ROS and oxidative stress or activate the programmed cell death (PCD). To prevent this copper-dependent oxidative burst, Cu^+^ must be chelated by intracellular cysteine-rich metallothioneins (MTs) or specific chaperones whose synthesis has a high energy cost, thus jeopardizing plant growth (Printz et al., 2016). In contrast, VPH peptides, particularly amino acids and small peptides that can be absorbed by plants (Colla et al., 2015), can complex not only Cu^+^, but also Cu^2+^, maintaining its redox capability, and facilitate Cu transport and allocation to Cu proteins, as also recently seen in medical studies on Cu-based chemotherapeutics (Peña et al., 2022).

Consistently, untargeted metabolomics unraveled a distinctive reprogramming of basil leaf metabolome in response to the different treatments, generally displaying a remarkable modulation of secondary metabolite concentrations.

The exposure of basil plant to Cu treatment induced significant changes in its alkaloid, terpenoid, and phenylpropanoid profiles, showing the most marked accumulation compared to the other treatments. This result can be interpreted as plant protective effect against physiological processes’ damage and oxidative stress, due to the excess presence of Cu. Several studies on many other plant species (*Brassica napus* L., *Brassica juncea* L., *Cucumis sativus* L., *Oryza sativa* L., and *Coriandrum sativum* L.) showed that plant exposure to substantial amount of Cu may result in increased ROS production—hydrogen peroxide (H_2_O_2_), superoxide anion radicals (O^2−^), and hydroxyl radicals (OH^−^)—causing damage to cellular constituents (Shaw and Hossain, 2013; Sunita and Shekhawat, 2016; Nair and Chung, 2017; Mosa et al., 2018; Alquraidi et al., 2019). ROS produced may cause lipid peroxidation, stimulating the production of highly active signaling compounds competent of triggering the production of secondary metabolites in the plant (Anjitha et al., 2021).

In this regard, it is well known that alkaloids represent key components of the plant defense system, being released by the plant to face stress conditions. In particular, the antioxidant activity of alkaloids includes the upregulation of ROS scavenging, the inhibition of ROS-producing enzymes, and the chelation of metals (Yoon et al., 2006). Lala (2020) reported a significant increase in the total content of alkaloid metabolites following Cu-based treatment in *Bacopa monnieri* L., accomplished by an increase in ROS markers. Moreover, enhanced production of alkaloids was found in *O. basilicum* L. and *Catharanthus roseus* L., respectively, following copper sulfate (CuSO_4_) and copper oxide (CuO) treatments (Pan et al., 2015; Nazir et al., 2021).

Likewise, plant production of terpenoids with antioxidant activities to cope and overcome abiotic stress is increasingly described in the scientific literature (Toffolatti et al., 2021). When exposed to the above threshold concentration of trace metals, plants can modify metabolic processes by synthesizing them (Anjitha et al., 2021). Kunwar et al. (2015) accounted significant changes in basil terpenoid composition by the addition of Cu, showing improved concentrations of linalool, chavicol, and methyl cinnamate when compared to the control (Kunwar et al., 2015). Interestingly, Hojati et al. (2017) investigated the response of *Tanacetum parthenium* L. to copper chloride dihydrate (CuCl_2_·2H_2_O) by associating stress-related responses with changes in terpenoids: low Cu (5 μM) supply increased the proportion of sesquiterpenes, both hydrocarbons and oxygenated, while elevated Cu levels reduced the total content of isoprenoids, by depressing the uptake of divalent cations, which are essential cofactors of enzymes involved in the biosynthesis of these metabolites.

In contrast, VPH-, Cu-VPH-, and VPH+Cu-VPH-treated plants displayed lower contents of alkaloids and terpenoids. Overall, we infer that the lower presence of these compounds following VPH supply is related to healthier plants with reduced levels of ROS. These results are in accordance with the recent findings of Sorrentino et al. (2021) where Trainer^®^ VPH treatment on *Arabidopsis thaliana* showed a clear downregulation of terpenoid compounds (Sorrentino et al., 2021). Likewise, Lucini et al. (2020) pointed out a general decrease of defense compounds, including alkaloids and terpenoids following the application of the same VPH on *Solanum lycopersicum* L. (Lucini et al., 2020).

Moreover, it is interesting to note that phenylpropanoid metabolites were markedly accumulated only in Cu-treated samples, since VPH+Cu-VHP induced just a weak increase and noteworthy downregulations were observed with VPH and Cu-VPH treatments. It is proven that the phenylpropanoid biosynthetic pathway is triggered under heavy metal stress, leading to the accumulation of phenolic compounds with ROS scavenging capacity (Park et al., 2022). Mira et al. (2002) extensively described flavonoid ability to exert pro-oxidant effects under Cu metal stress through the metal chelating process. This clearly explains our finding that the levels of phenylpropanoid and flavonoid compounds were found to be increased by Cu excess. Our results are in accordance with previous reports in which CuO nanoparticles enhanced total flavonoid content in *Stevia rebaudiana*, *Brassica nigra* L., and *O. basilicum* L. (Zafar et al., 2017; Javed et al., 2018; Nazir et al., 2021). Moreover, our study is coherent with the previous work of Sorrentino et al. (2021), which ascribed to the same VPH a significant downregulation of flavonoid compounds.

## 5 Conclusions

The widespread awareness of the impact of anthropogenic activities on the environment calls for careful and critical reflection on the agricultural sector’s future. The unconditional use of chemical fertilizers and pesticides is no longer sustainable, requiring growers to use innovative techniques that are increasingly eco-friendly. Cu, despite being a broad-spectrum fungicide and nutritionally essential element for plants, is a heavy metal that may accumulate and enter trophic chains. For this reason, its use with new formulations may be a necessary sustainable means of reducing dosages and increasing crop uptake and use efficiency. Regarding the mineral profile, the plants treated with Cu-VPH had the same copper concentration as those treated with inorganic copper. Still, at the same time, the use of Cu-VPH significantly improved the concentrations of K, Mg, and Fe, whereas VPH+Cu-VPH treatments, which showed half of leaf Cu concentration compared to Cu and Cu-VPH treatments, underwent a better Ca and Mn uptake capacity, and Cu and N use efficiency. It is likely that Cu integration as a cofactor in transporters of the photosynthetic electron transport chain, and that of Mn in the water splitting system, improved the maximum potential quantum efficiency of Photosystem II (Fv/Fm) and the electron transport flow under high light. At the same time, the carboxylation capacity of the plants and the synthesis and translocation of amides increased, thus enhancing the physiological and productive performance of Genovese basil and its fresh production compared to the other treatments and the untreated control. Finally, untargeted metabolomics pointed out increased concentrations of stress-related compounds (mainly alkaloids, terpenoids, and phenylpropanoids) following Cu supply, differently from VPH-Cu, VPH, and VPH+Cu-VPH treatments, which showed low levels of these compounds. This suggested the VPH ability to alleviate the negative effect of oxidative stress due to plant exposure to substantial amount of Cu. Therefore, in light of the promising results obtained in this study and with a view to enhancing agricultural sustainability, producers should promote the use of complex micronutrients combined with biostimulants.

## Data availability statement

The original contributions presented in the study are included in the article/**Supplementary Material**. Further inquiries can be directed to the corresponding authors.

## Author contributions

GC and YR: conceptualization and project administration. YR, PC, MC, LF, CE-N, PG, AF, BMM, MTC, LL, and GC: methodology, validation, formal analysis, investigation, and writing—original draft preparation. YR, PC, MC, LF, CE-N, PG, AF, BMM, MTC, LL, and GC: software. YR, PC, MC, LF, CE-N, PG, AF, BMM, MTC, LL, and GC: resources. YR, PC, MC, LF, CE-N, PG, AF, BMM, MTC, LL, and GC: writing—review and editing. YR, PC, LL, and GC: visualization. YR, PC, LL, and GC: supervision. YR, PC and GC: funding acquisition. All authors contributed to the article and approved the submitted version.
