# Supplementary material for: Copper boosts the biostimulant activity of a vegetal-derived protein hydrolysate in basil: morpho-physiological and metabolomics insights
Source: Front Plant Sci. 2023 Aug 24;14:1235686. doi: 10.3389/fpls.2023.1235686 (PMC10484225; doi:10.3389/fpls.2023.1235686)
Supplement: Supplementary file 2 [file Table_2.docx]

**Supplementary Table 1.** CIELab colorimetric parameters of basil leaves under untreated control, copper sulphate (Cu), copper complexed with amino acids and peptides (Cu-VPH), vegetal protein hydrolysate (VPH) and Cu-VPH enriched with VPH (VPH+Cu-VPH) treatments.

| Treatments | L | a* | b* | SPAD index |
| --- | --- | --- | --- | --- |
| Control | 45.63 ± 0.32 bc | –7.61 ± 0.12 c | 18.01 ± 0.74 b | 37.34 ± 0.34 b |
| Cu | 47.55 ± 0.14 a | –6.38 ± 0.13 ab | 14.50 ± 0.31 c | 37.58 ± 0.28 b |
| Cu-VPH | 47.59 ± 0.37 a | –6.03 ± 0.12 a | 13.38 ± 0.27 c | 39.28 ± 0.37 a |
| VPH+Cu-VPH | 44.61 ± 0.38 c | –8.77 ± 0.16 d | 21.68 ± 0.86 a | 40.25 ± 0.42 a |
| VPH | 46.21 ± 0.26 ab | –6.82 ± 0.09 b | 15.33 ± 0.56 bc | 39.21 ± 0.17 a |
| Significance | *** | *** | *** | *** |

*** significant at *p* ≤ 0.001. Different letters within each column indicate significant differences according to Tukey HSD test (*p* = 0.05). All data are expressed as mean ± standard error, *n* = 3.
